# Menorrhagia: an underappreciated problem in pre-menopausal women with systemic lupus erythematosus

**DOI:** 10.1177/0961203319851868

**Published:** 2019-05-27

**Authors:** C Wincup, T C R McDonnell, A Rahman

**Affiliations:** Department of Rheumatology, University College London, London, UK

Sir,

Anaemia is commonly seen in patients with systemic lupus erythematosus (SLE) and may be the result of haemolysis, drug treatment or anaemia of chronic disease.^1,2^ However, in the general population, the most common cause of anaemia is iron deficiency secondary to menstrual blood loss.^3^ A previous European-wide study of >4000 otherwise healthy pre-menopausal women found that symptomatic heavy menstrual bleeding (HMB or menorrhagia) affected 27% of those surveyed. This problem was further compounded by the fact that 46% of symptomatic patients had never consulted a healthcare professional regarding this problem.^4^ It is therefore surprising that, while 90% of patients with SLE are female, little is known with regard to the prevalence of menorrhagia.

In line with previously published studies, we conducted a ‘female health questionnaire’-based survey that sought to identify the rates of menorrhagia in female patients with SLE attending the Lupus Clinic at University College London Hospital, UK. Menorrhagia was defined as the presence of two or more out of the following four symptoms (as defined in previously validated studies^4,5^): (a) flooding through clothes or bedding, (b) the need to change sanitary products every 2 hours or less, (c) the need to double sanitary protection and (d) passing large blood clots. In addition, patients were asked to confirm the number of menstrual cycles experienced in the last 12 months and whether they had previously sought help from a healthcare professional for symptoms of menorrhagia. History of anaemia and of previous iron supplementation were also recorded.

A total of 116 patients fulfilling revised American College of Rheumatology classification criteria^6^ participated in the study. Forty-six patients were excluded in view of being post-menopausal (with no menstrual periods reported in the previous 12 months).

The remaining 70 pre-menopausal patients showed typical characteristics of our clinic population with mean (±SD) age 37.8 ± 7.1 years, mean (±SD) disease duration 16.8 ± 7.2 years and ethnicity 49% Caucasian, 20% Afro-Caribbean and 19% Asian. Although reduced rates of menstruation and amenorrhoea have previously been reported in SLE patients,^7,8^ these 70 patients reported a mean (±SD) of 10.7 (±6.3) menstrual cycles per year.

Menorrhagia (identified by at least two of the four symptoms outlined above) was seen in 49% of all pre-menopausal patients with lupus. This is significantly higher than the 27% reported at a population level.^4^ Patients with menorrhagia were significantly more likely to report a history of anaemia (*p* = 0.02) and previous treatment with iron supplementation (*p* = 0.04). These results are summarized in Table 1. Of the patients with menorrhagia, 41% had not consulted healthcare professionals regarding these symptoms.

**Table 1:** Prevalence of symptoms of heavy menstrual bleeding and comparisons between symptomatic and non-symptomatic patients

Total number of symptoms of HMB	% (n) of pre-menopausal patients (*n* = 70)			
0	30% (21)			
1	21% (15)			
2	7% (5)			
3	11% (8)			
4	30% (21)			
*Questionnaire Results*	*All pre- menopausal patients (*n* = 70)*	*Non-symptomatic: patients with <2/4 symptoms (*n* = 36)*	*Symptomatic: patients with ≥2 symptoms (*n* = 34)*	p-*value*
Total number of menstrual periods in last 12 months ± SD	10.7 ± 6.3	10.5 ± 4.0	11.0 ± 8.1	NS
Using OCP % (n)	9% (6)	11% (4)	6% (2)	NS
Using implant/coil % (n)	7% (5)	3% (1)	12% (4)	NS
History of anaemia % (n)	63% (44)	50% (18)	76% (26)	0.02*
Previous iron supplementation % (n)	71% (50)	61% (22)	82% (28)	0.04*
Oral % (n)	61% (43)	50% (18)	74% (25)	0.04*
Intravenous % (n)	19% (13)	19% (7)	18% (6)	NS

SD, standard deviation; OCP, oral contraceptive pill; HMB, heavy menstrual bleeding *significant at <0.05.

These findings suggest that menorrhagia is common in women with SLE and is more prevalent than in the general population, thus potentially representing an underappreciated cause of iron deficiency and anaemia. Many of these patients do not report symptoms of menorrhagia to healthcare professionals. Therefore, it is important to consider asking about this issue routinely, in particular in patients with lupus who are anaemic without any obviously apparent alternative cause.
